# Transcriptomic analysis of hepatocellular carcinoma reveals molecular features of disease progression and tumor immune biology

**DOI:** 10.1038/s41698-018-0068-8

**Published:** 2018-11-15

**Authors:** K. Okrah, S. Tarighat, B. Liu, H. Koeppen, M. C. Wagle, G. Cheng, C. Sun, A. Dey, M. T. Chang, T. Sumiyoshi, Z. Mounir, C. Cummings, G. Hampton, L. Amler, J. Fridlyand, P. S. Hegde, S. J. Turley, M. R. Lackner, S. M. Huang

**Affiliations:** 1 Department of Biostatistics, Genentech, 1 DNA Way, South San Francisco, CA 94080 USA; 2 Department of Oncology Biomarker Development, Genentech, 1 DNA Way, South San Francisco, CA 94080 USA; 3 Department of Research Pathology, Genentech, 1 DNA Way, South San Francisco, CA 94080 USA; 4 Department of Research, Genentech, 1 DNA Way, South San Francisco, CA 94080 USA

## Abstract

Hepatocellular carcinoma (HCC) develops in the context of chronic inflammatory liver disease and has an extremely poor prognosis. An immunosuppressive tumor microenvironment may contribute to therapeutic failure in metastatic HCC. Here, we identified unique molecular signatures pertaining to HCC disease progression and tumor immunity by analyzing genome-wide RNA-Seq data derived from HCC patient tumors and non-tumor cirrhotic tissues. Unsupervised clustering of gene expression data revealed a gradual suppression of local tumor immunity that coincided with disease progression, indicating an increasingly immunosuppressive tumor environment during HCC disease advancement. IHC examination of the spatial distribution of CD8+ T cells in tumors revealed distinct intra- and peri-tumoral subsets. Differential gene expression analysis revealed an 85-gene signature that was significantly upregulated in the peri-tumoral CD8+ T cell-excluded tumors. Notably, this signature was highly enriched with components of underlying extracellular matrix, fibrosis, and epithelial–mesenchymal transition (EMT). Further analysis condensed this signature to a core set of 23 genes that are associated with CD8+ T cell localization, and were prospectively validated in an independent cohort of HCC specimens. These findings suggest a potential association between elevated fibrosis, possibly modulated by TGF-β, PDGFR, SHH or Notch pathway, and the T cell-excluded immune phenotype. Indeed, targeting fibrosis using a TGF-β neutralizing antibody in the STAM™ model of murine HCC, we found that ameliorating the fibrotic environment could facilitate redistribution of CD8+ lymphocytes into tumors. Our results provide a strong rationale for utilizing immunotherapies in HCC earlier during treatment, potentially in combination with anti-fibrotic therapies.

## Liver cancer: Poor tumor immunity linked to fibrosis

Anti-fibrotic drugs could help make patients with liver cancer more responsive to immune-targeted therapies. A team led by Shih-Min Huang and Mark Lackner from Genentech in South San Francisco, CA, USA, analyzed gene expression profiles in tumor samples taken from 98 people with hepatocellular carcinoma, the most common form of liver cancer. The expression analysis revealed a persistently down-modulated immune microenvironment—and the more advanced the disease, the stronger the immune suppression. The researchers also saw an association between increased expression of genes involved in fibrotic tissue scarring and diminished immune function. They therefore administered an antibody drug that blocks a key growth factor implicated in fibrosis to a mouse model of the disease. These animals showed enhanced immune function, raising the possibility of using a similar strategy in patients undergoing immunotherapy for liver cancer.

## Introduction

Hepatocellular carcinoma (HCC) is the second most common cause of death from cancer worldwide and has an extremely poor prognosis.^1^ It is known that the majority of HCC arises from chronic liver fibrosis or cirrhosis caused by constant cycles of injury and regeneration originating from various etiologies, including HBV/HCV viral infection, non-alcoholic fatty liver disease, excessive alcohol intake, and aflatoxin consumption.^2–4^ The prolonged timeline of disease progression from the inception of chronic liver injury to the subsequent exhibition of cirrhosis and the eventual manifestation of malignancy, along with the heterogeneous nature of HCC, add complexity to dissecting the biology of this disease.^3^ Recently, publications focusing on whole-genome or whole-exome sequencing have shed light on HCC disease biology from the perspective of genetic alterations.^5–16^ These studies have implied links between genetic mutations to specific risk factors and recurrently altered pathways, thereby offering therapeutic hypotheses for further clinical experimentation.

Nevertheless, as a disease with a lengthy time frame for development, HCC progression can also be significantly influenced by multiple additional biological processes including epigenetics and the immune microenvironment in tumor tissues.^17–19^ To provide further insight into the HCC progression landscape and biology, a comprehensive whole transcriptomic analysis of specimens representing different disease stages might offer higher resolution as to the underlying mechanisms of progression.

Given the indolent nature of HCC, the intimate interactions between the tumor environment and malignant cells may be of significance during disease progression. Close associations between fibrosis and epithelial–mesenchymal transition (EMT) have been described previously.^20^ Furthermore, the contribution of EMT to the maintenance of suppressive tumor immune environment is well documented.^21,22^ The liver plays a major role in controlling metabolism, detoxification, and nutrient storage, all of which contribute to a somewhat immunosuppressed environment. Thus, boosting HCC tumor immunity through modulation of the immediate immune contexture might be an effective way to combat this disease.^23–25^

Current therapeutic interventions in HCC have focused largely on targeting tumor cells through surgery, radiofrequency ablation, and local or systemic administration of chemotherapies, especially in the early stages of HCC.^3^ Nevertheless, reoccurrence is still a profound issue in extending patient survival.^3,26^ Sorafenib is the only FDA approved therapy recommended as first-line treatment in the metastatic setting.^27^ However, most patients progress rapidly on sorafenib, suggesting a need for additional therapeutic options for HCC patients. Recently, the PD-1 checkpoint inhibitor nivolumab showed significant therapeutic benefit in HCC and was approved for this indication as a second line treatment option in the metastatic setting.^28–31^ Specifically, the updated objective response rate measured by RECIST v1.1 was 20%, with a 9-month overall survival rate of 74%.^30,31^ Interestingly, the clinical response did not appear to correspond with the expression of PD-L1 on tumor cells, highlighting the importance of the immune composition of HCC tumors and the surrounding stromal milieu. These exciting data provide a novel treatment option for patients with advanced HCC. However, combination therapy will likely be needed to achieve further improvements in efficacy, thus raising the question of which therapeutic options and combinations may be most beneficial.

Here, we investigated prominent biological features that are associated with HCC disease progression by probing whole-transcriptome RNA-Seq data from a collection of cirrhotic and HCC tumor specimens (clinically staged from T1N0M0 to T3N0M0 with TNM staging system). Our data shows a persistently down-modulated tumor immune environment, implying increasingly inferior tumor immunity in more advanced stages of HCC. Additional analyses exploring the biological determinants of the spatial distribution of CD8+ T cells indicated a potential association between increased levels of extracellular matrix/fibrotic contexture/EMT and the diminished tumor penetrance of CD8+ T effector cells. Further, administration of α-TGF-β appeared to ameliorate the fibrotic milieu of STAM™ model of murine HCC and lead to enhanced distribution of CD8+ T cells, thereby highlighting the potential benefits of combining anti-fibrotic therapies with immune checkpoint inhibitors to treat HCC.

## Results

### A progressively downregulated immune gene profile is associated with HCC disease progression

**Fig. 1:**
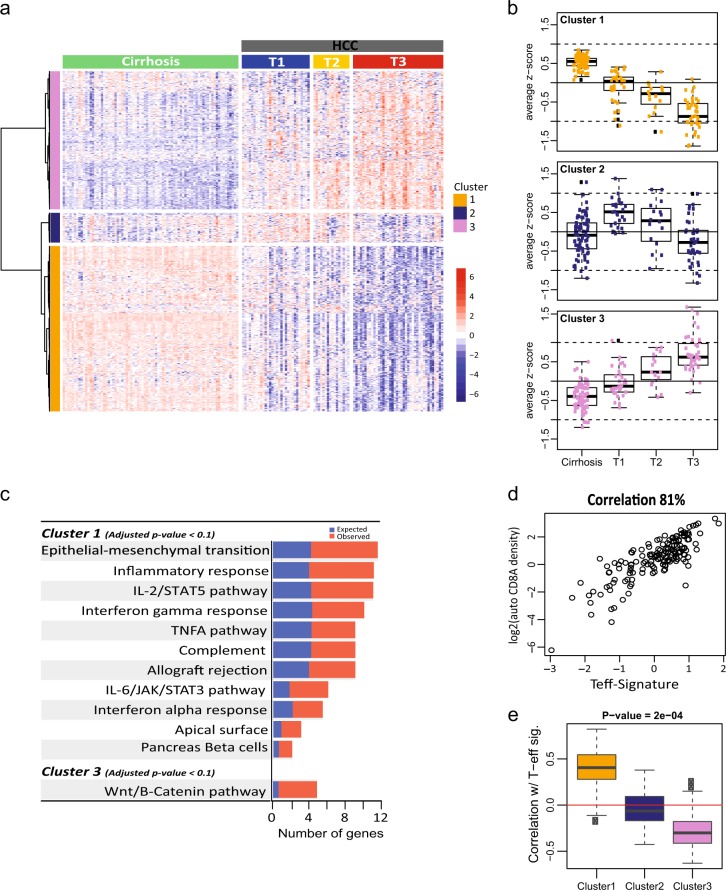
Three distinct gene sets are associated with HCC disease stage and immune environment. **a** Three distinct gene clusters whose expression was associated with different disease stages. Clusters were identified by analysis of variance hypothesis testing. Clustering was done for all patients based on all 15,524 genes (transcriptome), with each patient profile (column) labeled with tissue classification (cirrhosis or HCC tumor) and tumor stages (T1–T3). **b** The gene signature (average of *z*-scores of genes in each cluster) shows a clear association between stage and signature. Top panel: Cluster 1 genes show a monotonically decreasing trend with progressive disease stages. Middle panel: expression of genes in Cluster 2 was higher in T1 than in cirrhotic tissues but decreased with more advanced stages of HCC. Bottom panel: Cluster 3 genes show a steady increase in expression with progressive disease stages. **c** Geneset enrichment analysis (GSEA) using Hallmark Gene Sets in Molecular Signature Database was used to identify the top-scoring genes and pathways in Clusters 1 and 3. Cluster 1 was overrepresented by Hallmark gene sets describing immune response while Cluster 3 was dominated by Hallmark gene sets associated with WNT pathway activation. None of the Hallmark gene sets were significantly enriched in Cluster 2 (not shown). **d** Aggregated expression of genes describing T effector signature (T-eff), including GZMA, GZMB, PRF1, EOMES, and CD8A, was significantly correlated with Cluster 1, *p*-value < 0.001. **e** Correlation between T-eff gene signature (distilled from RNA-Seq dataset) and CD8A expression (measured by immune histopathology). Strong directional relationship can be detected between the two assessments

The majority of HCC incidences arise from liver cirrhosis following a progressive course of disease advancement.^2^ We sought to investigate the transcriptomic profile of HCC samples and non-tumor cirrhotic tissues with an emphasis on discovering novel and unique molecular features associated with the progression of this deadly disease. To this end, whole transcriptome RNA-Seq was performed on 98 clinically graded human HCC tumor samples (tumor staging: T1N0M0 to T3N0M0) and non-tumor cirrhotic tissues obtained from 78 of the aforementioned HCC patients (patient classification info; Table 1 and Supplementary Table S1). Of note, the tumor content of the majority of HCC samples fell within 60–80% and macrodissection was performed prior to RNA extraction (Supplementary Fig. S1A). Initial principal component analysis confirmed that the cirrhotic specimens were indeed molecularly distinct from the HCC tumor samples (Supplementary Fig. S1B). Subsequent analysis of variance hypothesis testing was performed to retain genes whose expression levels were associated with tumor stages (T1, T2, or T3), but independent of the ordering of tumor stages. Interestingly, the analysis illustrated that most specimens of stage T1 remain closely related to cirrhotic tissues illustrated by highly similar transcriptomic profiles. Whereas those of T2 and T3 stages are largely distant from non-tumor cirrhotic tissues, corroborating the epidemiological observation that majority of HCC cases arise from the preceding diagnosis of cirrhosis (Fig. 1a).

**Table 1:** Characteristics of the 98 liver cancer samples summarized based on demographics, etiology, and tumor stage information

Patient samples^a^	HCC (*n* = 98)
**Demographics**	
Sex (*n* (%))	
Male	83 (85)
Female	15 (15)
Age (*n* (%))	
≤50	31 (32)
>50	67 (68)
Region (*n* (%))	
Asia	98 (100)
Etiology (*n* (%))	
HBV	76 (78)
HCV	2 (2)
Non-HBV/HCV	20 (20)
**Tumor burden**	
TNM stage (*n* (%))	
Primary tumor (T)	
T1	30 (31)
T2	16 (16)
T3	40 (41)
TX	12 (12)

^a^78 HCC samples were provided with paired cirrhosis specimens

TX: primary tumor cannot be assessed, T1: solitary tumor without vascular invasion, T2: solitary tumor with vascular invasion or multiple tumors, none > 5 cm, and T3: multiple tumors > 5 cm

The resulting genes yielded three distinct gene clusters with transcript levels directionally associated with disease stages (Fig. 1a, see Materials and methods for the detailed statistical methods and Appendix 1 for gene signature content). For example, gene Cluster 1 illustrated a continuous downward expression from T1 to T3 while Cluster 3 exhibited upward expression from T1 to T3 (Fig. 1a, b). Cluster 2 demonstrated an increased expression level in T1, but subsided from T2 to T3 (Fig. 1a, b). Subsequently, we performed an unbiased gene set enrichment analysis (GSEA) on these gene clusters using the Hallmark Gene Sets from the Molecular Signature Database.^32,33^ Interestingly, in Cluster 1, we found an overrepresentation of Hallmark Gene Sets describing transcriptional events directionally up-regulated by the indicated immune stimulations, such as inflammatory response, IL2, IFNg, allograft rejection, etc. (Fig. 1c). On the other hand, Cluster 3 was highlighted by genes representing WNT pathway activation such as DVL1/2, and conserved downstream transcriptional targets such as AXIN2, NKD1, RNF43, and ZNRF3 (Fig. 1c, gene list in Appendix 1). These results are in line with recent findings that increased WNT pathway activity can exert an immunosuppressive effect on the tumor microenvironment.^34^ Of note, no significant biological features were found to be associated with Cluster 2 (gene lists are provided in Appendix 1). Our findings, therefore, indicate a relationship between the deteriorating immune contexture and progressively worsening liver cancer.

Cytotoxic T effector cells have been shown to be the main source of anti-tumor immune response.^23,35^ The aggregated transcript levels of genes (Teff signature) representing Granzyme A (GZMA), Granzyme B (GZMB), Perforin 1 (PRF1), Eomesodermin (EOMES), and CD8A can be closely associated with density of CD8+ T cells measured by IHC. In fact, we found a strong correlation (81%) between the Teff signature and CD8A expression evaluated by IHC in HCC samples (Fig. 1d). Interestingly, the levels of Teff signature were most strongly correlated with the expression of genes in Cluster 1, than with genes in either Cluster 2 or 3, further confirming the increasingly diminishing tumor adaptive immune environment in HCC (Fig. 1e). As previously mentioned, the tumor environment in HCC is considered to be less immunogenic.^25^ However, to our knowledge, this is the first demonstration of a progressively eroding tumor immune environment corresponding to the more advanced stages of HCC tumors.

### Exclusion of CD8+ T lymphocytes is associated with the magnitude of fibrotic/ECM/EMT transcript signals

**Fig. 2:**
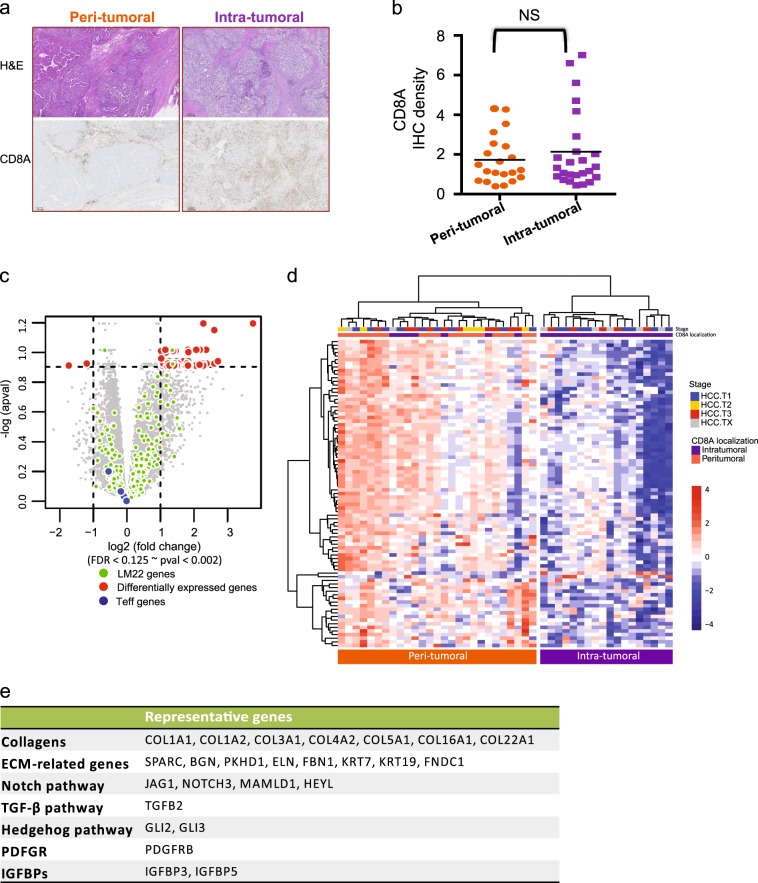
CD8 excluded immune phenotype is associated with genes representing fibrosis and cirrhosis process. **a** Hematoxylin and eosin stain (top) and immunohistology of two immune phenotypes in HCC samples. IHC was performed on FFPE sections stained for CD8 positive T lymphocytes. The two peri-tumoral and intra-tumoral classifications were based on spatial distribution and infiltration of CD8 cells within the malignant hepatic cells and stromal content. Scale bar = 300 µm. **b** Depiction of CD8 cells’ density score determined by image analysis of IHC slides as compared between the two immune phenotypes. **c** Genes differentially expressed between peri-tumoral and intra-tumoral HCC samples. A set of *n* = 85 genes (red dots: herein called CTL-Ex) were found to be most significantly upregulated in peri-tumoral specimens. Green dots represent genes in the LM22 leukocyte gene signature matrix.^53^ **d** A heatmap representing intra-tumoral and peri-tumoral samples demonstrating that CTL-Ex genes expression was not associated with disease stages. TX denotes samples with unknown tumor stage. **e** A summary of some of the genes in CTL-Ex, including collagens and ECM-related genes

In general, tumors presenting intra-tumoral CD8+ cytotoxic T lymphocytes (CTL) are often classified as “inflamed”, while those featuring peri-tumoral CD8+ CTL localization are collectively named “excluded-infiltrates”.^36^ The presence of CD8+ CTL has been shown to be associated with response to the checkpoint inhibition by α-PD-1/PD-L1, suggesting the significance of pre-existing CD8+ CTL in tumors.^37^ Thus, the localization of CD8+ CTL appears to be another determinant of tumor immunity architecture. These published observations led us to investigate the tumor immune environment as described by the spatial distribution of CD8+ CTL in HCC tumor specimens. As shown in Fig. 2a, two distinct sub-populations featuring either “intra-tumoral” or “peri-tumoral” localization of CD8+ CTL emerged after assessing CD8 IHC images, representing 27% and 23% of the total stained samples, respectively (see Supplementary Table S2 for CD8+ CTL localization classification for all HCC samples). Protein and mRNA expression levels of CD8A were relatively indistinguishable between the two populations (Fig. 2b; Supplementary Fig. S2). Through differential gene expression analysis, we attempted to explore potential molecular mechanisms of T cell exclusion. At the cut-off of false discovery rate (FDR) < 0.125 and ≥2-fold change, 85 genes were found to be upregulated in specimens showing peri-tumoral phenotype, which we collectively named Cytotoxic T Lymphocytes Exclusion Gene Set (CTL-Ex) (Fig. 2c, d, Materials and methods). The expression of CTL-Ex genes does not appear to be closely associated with disease stages (Fig. 2d). However, the CTL-Ex gene signature most strongly represents the biological mechanisms that are involved in Hepatic Fibrosis/Hepatic Stellate Cell Activation (Supplementary Fig. S3). In addition, the top-scoring genes in this signature include collagens and components of extracellular matrix, along with constituents of pathways known to be positively regulating fibrosis in various organs (e.g., TGF-β2, PDGFRβ, GLI1, GLI2, JAG1, NOTCH3, MAMLD1, and HEYL, representing TGF-β, PDGFR, SHH, and NOTCH pathways respectively^33^) (Fig. 2e). Since all HCC specimens were obtained from patients that were clinically diagnosed with either cirrhosis or fibrosis, it was rather surprising that the biological signature of liver fibrosis was elevated in samples with peri-tumoral distribution of CD8+ T cells. That said, diagnostic methods for fibrosis in the clinic are not standardized and have a relatively small dynamic range. It remains plausible that fibrosis activation as measured by gene expression profiling may offer a larger dynamic range that can more effectively capture correlations between fibrosis and the differential penetrance of CD8+ T lymphocytes into tumors.

### A subset of Cytotoxic T Lymphocytes Exclusion Gene Set (CTL-Ex) is conserved in various indications

**Fig. 3:**
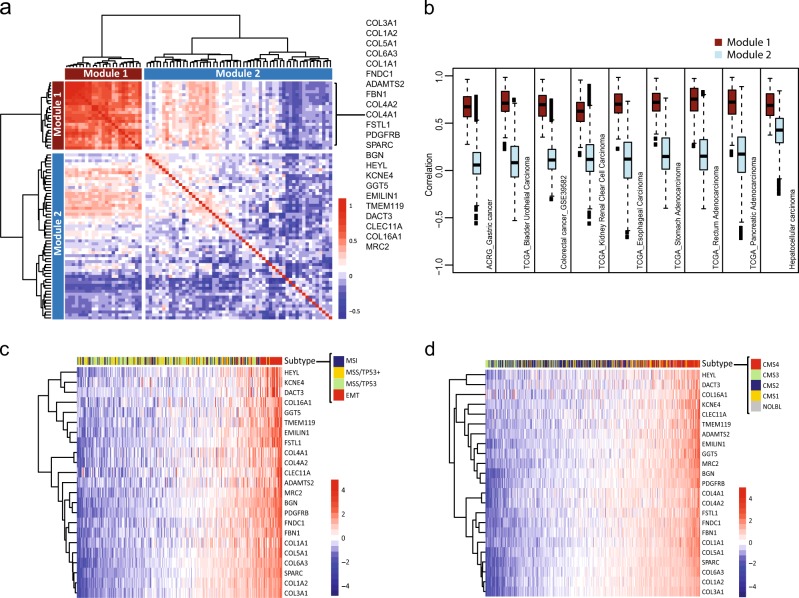
CD8 exclusion gene signature has distinguishing power across multiple indications. **a** Minimum reference correlation matrix summarizing the pairwise correlations amongst CTL-Ex genes across multiple indications. A subset of *n* = 23 highly correlated genes cluster closely together (Module 1). Genes associated with components of ECM, EMT, and fibrosis represented the components of Module 1. Module 2 represents the remaining 62 genes identified as CTL-Ex but not consistently correlated across indications. **b** Box plot depicting the extent of pairwise correlations for Modules 1 and 2 across multiple cancer types. High correlation (median pairwise correlation above 0.5) was maintained for Module 1 for all indications tested while Module 2 remained relevant in HCC. **c** Module 1 genes identify the EMT subtype in gastric cancer (based on data and subtyping of published data.^40^ **d** Module 1 gene signature in colorectal cancer data is associated with the mesenchymal molecular subtype as identified according to the previous classification by CRC subtyping^49^

Chronic tissue fibrosis followed by enhanced production of ECM and soluble factors can suppress local tumor immunity, prevent CTL infiltration, and promote tumor initiation and growth.^38^ Therefore, reversing ECM accumulation caused by fibrosis may enhance tumor-specific immunity across indications.^39^ To assess the general applicability of CTL-Ex, we first attempted to extract the minimal gene set from CTL-Ex signature derived from our HCC samples. With further correlation analysis, we identified a collection of 23 genes that clearly forms a distinct cluster (Module 1) from the remaining 62 genes (Module 2) (Fig. 3a; Materials and methods). Of note, Module 1 mostly represented collagens and ECM components (Fig. 3a). The extent of correlation for Module 1 was relatively consistent among all indications tested, including gastric, colorectal, pancreatic, esophageal, bladder, and renal cancers, whereas Module 2 only maintained a degree of correlation in HCC (Fig. 3b). In addition, utilizing published information on a comprehensive subtyping effort in gastric cancer by Cristescu et al., we demonstrated that Module 1 readily identifies EMT subtype that was initially classified by a 149 EMT upregulated gene signature^40^ (Fig. 3c). In colorectal cancer, a recent concerted effort in molecular classification carried out by the CRC Subtyping Consortium yielded CMS1–4 subtypes in which the CMS4 subtype was dubbed “mesenchymal”, reflecting the biology of stromal infiltration, TGF-β activation, and angiogenesis. In addition, this subtype also conferred worse relapse-free and overall survival in patients. Similarly to gastric cancer, Module 1 also easily distinguished the majority of CMS4 patients in this study^41^ (Fig. 3d). Another recent study in metastatic urothelial carcinoma showed that lack of response to checkpoint inhibitor therapy was associated with increased TGFβ signaling in fibroblasts in the tumor microenvironment.^42^ These observations further corroborate that the fibrosis/EMT/ECM biological signals represented by CTL-Ex signature may be generalizable across different tumor types (Fig. 2e).

**Fig. 4:**
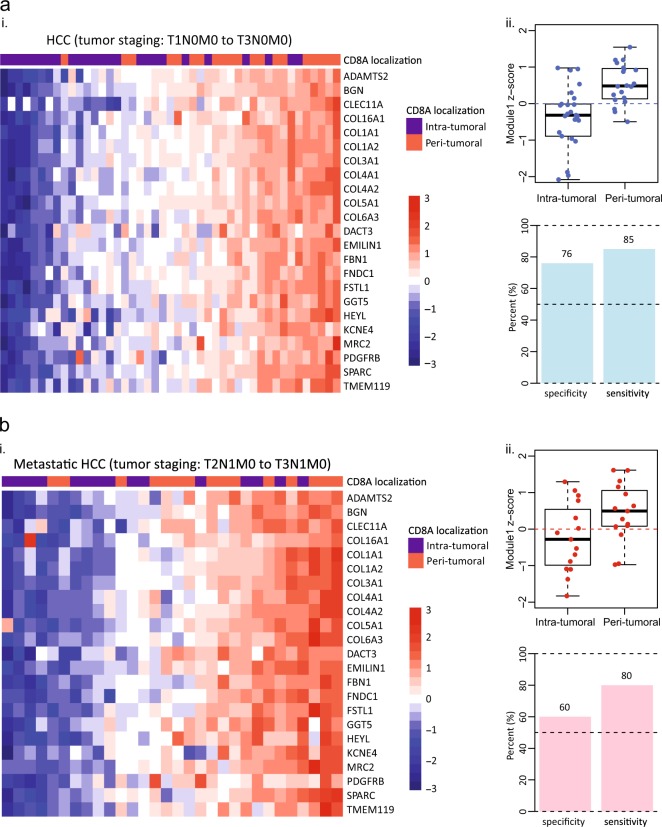
Intra-tumoral and peri-tumoral immune phenotypes are discernable using Module 1 gene signature. **a** (**i**) Heatmap demonstrating Module 1 gene’s utility in discriminating between the two distinct intra-tumoral and peri-tumoral immune phenotypes. (**ii**) Specificity and sensitivity of Module 1 when re-applied to HCC gene expression cohort. **b** (**i**) Module 1 gene signatures evaluated in an independent cohort of metastatic HCC samples. (**ii**) Specificity and sensitivity of Module 1 when applied to the independent metastatic HCC cohort

Subsequently, we examined the relationship of Module 1 to the localization of CD8+ T cells and re-applied Module 1 to the current cohort of HCC specimens (including all T1–T3 samples). The analysis illustrated that Module 1 could indeed reasonably distinguish specimens with intra-tumoral or peri-tumoral CD8+ T cell localization with statistical significance (Fig. 4a(i & ii); 76% specificity and 85% sensitivity). To further confirm this observation, a validation study was conducted utilizing an independent cohort of metastatic HCC specimens (disease staging: T2N1M0 to T3N1M0) (Supplementary Table S3 and Materials and methods). Both gene expression analysis and CD8 IHC readings were performed independently. The outcome of this validation study further demonstrated a potential causal relationship between high Module 1 expression and the peri-tumoral localization of CD8+ T cells (Fig. 4b(i & ii); 60% specificity and 80% sensitivity). Together, these findings strongly suggest that a fibrotic environment may prevent the distribution of CD8+ T cells in tumors.

### Blocking TGF-β can ameliorate the fibrotic state in HCC and promote CTL distribution

**Fig. 5:**
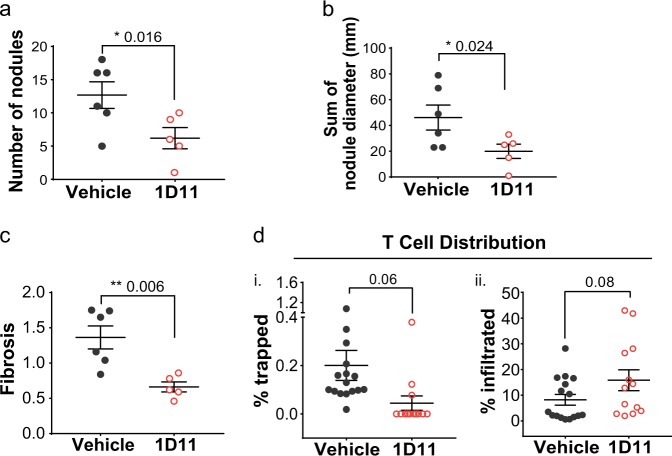
Inhibiting TGF-β signaling has anti-fibrotic effects in vivo. **a** Significant reduction in total number of nodules in the liver of HCC mouse model at the end of study after 6 weeks treatment with 1D11 (10 mg/kg) when compared to vehicle control treated animals. **b** Tumor size presented as the sum of nodule diameter (mm) in HCC mice treated with 1D11 when compared to vehicle control treated HCC animals. **c** Changes in the degree of hepatic fibrosis in a mouse model of HCC. Data points represent the percent positive fibrotic area quantified using image analysis of light microscopy images per mouse in vehicle control and 1D11-treated groups. **d** Quantification of the percentage of (**i**) trapped and (**ii**) infiltrated CD8+ T cells following 6 weeks of 1D11 treatment per malignant HCC nodule as contrasted by vehicle control animals. Trapped T cell immune phenotype was found more commonly in control group. In contrast, after 1D11 treatment to inhibit TGF-β, a more T cell infiltrated immune phenotype was observed. Bars represent mean with SEM. *p < 0.05, **p < 0.01, ns not statistically significant

Earlier, our analysis revealed that CTL-Ex immune exclusion signature was enriched by cellular components positively regulated by active transforming growth factor beta (TGF-β) pathway. Upregulation of TGF-β, a master regulator of fibrosis, has been shown to be associated with proinflammatory and profibrotic activities and to be inhibitory to checkpoint blockade.^42–44^ Here, we first examined the preclinical activity of a TGF-β neutralizing antibody (1D11) on activated human myofibroblast-like hepatic stellate cells (HStec) in vitro. HStec cells were cultured in the presence of increasing doses of 1D11 for 72 h and cell growth inhibition was determined. We found that 1D11 did not have a considerable adverse effect on the viability and growth of HStec cells during this time frame (Supplementary Fig. S4A). However, using ELISA to detect soluble Type I Collagen, we showed that treatment of HStec cells with 1D11 could lead to a dose-dependent downregulation of collagen secretion (up to 22% reduction, *p*-value = 0.004, Supplementary Fig. S4B). Notably, in vitro, 1D11 did not appear to be enhancing or to be cytotoxic toward activated T cell subpopulations (Supplementary Fig. S4C). Subsequently, we tested whether 1D11 is capable of remodeling the fibrotic tumor microenvironment and altering the localization of cytotoxic T cells within the tumor region. To this end, we examined the activities of 1D11 in vivo using a STAM™ mouse model for fibrotic disease and liver carcinogenesis. STAM™ is a model for non-alcoholic steatohepatitis (NASH) and HCC, created by a combination of chemical and dietary interventions in mice with a non-genetic C57BL/6 background (see Materials and methods). For this study, male mice were subjected to a single streptozotocin injection after birth and a high fat diet after 4 weeks of age which progressed to HCC around week 17 (evident by H&E staining, data not shown). These mice were then randomized and were treated with either anti-TGF-β (1D11; 10 mg/kg, twice a week), or vehicle control for 6 weeks. At the end of the study, tumor samples were harvested from the livers of animals for further analysis. Significant changes in tumor burden, represented by a decrease in the number of tumor nodules (Fig. 5a) and tumor size (Fig. 5b) were observed in mice that received 1D11. In addition, we also found that mice treated with 1D11 had reduced fibrosis as measured by Sirius Red staining when compared to the vehicle control group (Fig. 5c and Supplementary Fig. S5A: representative light microscopy images). To visualize and quantitate the changes in CD8+ T cell localization with or without 1D11 treatments, we utilized an α-CD8A-Masson’s trichrome double stain in IHC. Trichrome staining was used to distinguish collagenous fibrotic clusters within tumor tissue, whilst the distance of T cells relative to these fibrotic deposits was used to define two distinct T cell populations namely: trapped (T cells overlapping and residing <5 µm from fibrous deposits) and infiltrated (T cells found >5 µm away from fibrous deposits) (Supplementary Fig. S5B & C). Although the overall number of T cells remained somewhat similar in control and 1D11-treated animals (data not shown), the spatial distribution of CD8+ T cells within the tumor area was different between the two groups. Exposure to 1D11 increased the percentage of infiltrated CD8+ T cells in the tumor, whereas vehicle-treated animals displayed predominantly T cells that were confined within or found in close proximity (<5 µm) to the fibrotic clusters. Quantitative comparison of the percentage of T cells revealed an average 3.9-fold reduction in the extent of trapped T cells (Fig. 5d(i), *p*-value = 0.06), and a 1.9-fold increase in the percentage of infiltrated T cells (Fig. 5d(ii), *p*-value = 0.08) following 1D11 treatment. Though not statistically significant, the overall data supports a trend where anti-TGF-β intervention could effectively restructure the highly fibrotic microenvironment in HCC and allow T cell infiltration within the tumor.

## Discussion

HCC typically develops as a consequence of persistent and long-term chronic liver injuries followed by progression to severe fibrosis and cirrhosis.^3^ In this article, we took unique approaches to survey the transcriptomic landscape of HCC. By analyzing whole transcriptome RNA-Seq data originating from clinically graded HCC specimens and cirrhotic tissues in an unbiased manner, we uncovered a disease continuum manifested by gene expression signatures that correspond to disease progression and declining tumor immune contexture. Our analysis identified three distinct gene sets with expression levels directionally associated with HCC disease stages. These findings provide important insight into the molecular basis of HCC disease progression.

Recent studies have identified a largely immunosuppressive environment in the liver, which may contribute significantly to HCC progression.^35^ Of three gene clusters that we observed to be associated with disease staging (T1N0M0–T3N0M0), we found that expression of Cluster 1, overrepresented by genes that are upregulated in adaptive immunity according to GSEA, continuously declines as HCC advances. The fact that the Teff gene signature was more correlated with Cluster 1 further corroborated the GSEA analysis. Together, these results suggest a gradual erosion of tumor immunity that correlates with HCC advancement and may negatively affect the anti-tumor immune responses. However, it is understandable that by applying “bulk” RNA-seq approach, where various cell types are interrogated altogether, we may have negatively affected the resolution in precisely describing the cell source of Cluster 1 signature. Though we showed that Cluster 1 is more correlated with Teff gene signature (Fig. 1e), which is generally correlated with tumor infiltrated lymphocytes (TIL),^47^ it is difficult to pinpoint the immune and non-immune components in tumors contributing to Cluster 1 signature. With further observation of the current study, it might be pertinent to execute a follow-up experiment that entails obtaining fresh tumor tissues from different HCC stages, dissociating and isolating immune cells from non-immune cells with flow cytometry, followed by RNA-seq to enhance resolution of characterizing different cell types within the tumor to assess how they are associated with Cluster 1 gene signature.

WNT pathway activation in tumors has been shown to contribute to the maintenance of the immunosuppressive environment.^34^ As a developmental pathway, WNT downstream transcriptional targets are usually conserved and can reflect the extent of signal intensity.^45^ Strikingly, the Cluster 3 gene set contains not only conserved transcriptional targets such as AXIN2, NKD1, RNF43, and ZNRF3, but also the upstream component DVL2 (Fig. 1c, d), strongly implicating a gradual activation of WNT pathway through HCC advancement that may also contribute to the continuous deterioration of tumor immune contexture. In addition to immunosuppression, WNT activity within the tumor environment can also support the maintenance of stem-cell-like features and invasiveness in cancer cells.^46^ Especially, EMT has been shown to be partly mediated through the activation of the WNT pathway.^46^ Interestingly, our analysis indicated a positive correlation between an increase in the expression of genes representing WNT pathway activation and tumor stage, therefore possibly also implicative of processes involved in EMT activation.

Localization of CD8+ CTLs may be another key determinant in mounting immune response against tumors. We visualized CD8+ CTLs in our HCC samples using IHC staining against CD8A. Two distinct populations of tumors with either intra-tumoral (27% of all) or peri-tumoral (23% of all) CD8+ CTL localization could be established. The intensity of CD8A expression levels was similar in both populations. In peri-tumoral specimens, we identified a group of 85 genes that showed differential expression at the transcription level and which we collectively named CTL-Ex. We found that expression of CTL-Ex significantly overlaps with hepatic fibrosis/hepatic stellate cell activation gene set and is enriched with collagens, ECM components, and pro-fibrotic signaling genes. This is the first data which to our knowledge identifies a correlation between the molecular signature of the fibrotic state and the extent of CD8+ CTLs penetrance into the tumor in HCC. Importantly, within the CTL-Ex gene signature we identified a specific module containing 23 genes (i.e., Module 1) that maintains a high degree of correlation in multiple solid tumors, in addition to HCC. The 23 genes were predominantly collagens and ECM proteins, further signifying the possibility that these gene products may serve as a physical barrier to prevent CD8+ CTL infiltration. Utilizing Module 1, we easily identified the EMT subtypes from the ACRG gastric cancer study and the CMS4 subtype in the CRC study by the CRC Subtyping Consortium.^41^ Furthermore, we showed that Module 1 expression is associated with localization of CD8+ CTL not only in an exploratory cohort, but also in a validation cohort (Fig. 4a, b). These observations prompted us to ask whether disrupting the collagen/ECM structure could lead to the decrease in trapped CD8+ CTL. In this context, the TGF-β signaling, as a key regulator of fibrosis, represents an emerging therapeutic target.^47^ Here, we investigated the preclinical activities of neutralizing antibody 1D11 in directly targeting TGF-β and its pro-fibrotic functions in hepatic stellate cells (HStecs), the main source of activated myofibroblasts during liver injury. In vitro, we measured a dose-dependent decrease in collagen secretion by HStecs when cultured in the presence of 1D11. When used at the indicated dosages and length tested here, 1D11 was not cytotoxic toward hepatic cells or immune cells (including CD4+ and CD8+ T lymphocytes), implicating its utility in regressing fibrosis-induced collagen deposits in the microenvironment while leaving CTL intact. Notably, in vivo administration of 1D11 induced potent anti-tumor responses in a STAM™ model of HCC. When compared to vehicle control group, we observed a reduction in tumor burden in the liver of 1D11-treated animals, accompanied by considerable regression of fibrosis, evident by diminished collagen deposition. In addition, the percent of trapped CD8+ CTL showed a trend towards decreases in the 1D11-treated group with a concurrent increase in infiltrated CD8+ CTL. A recent study in urothelial carcinoma found that therapeutic co-administration of TGFβ-blocking and anti-PD-L1 antibodies reduced TGFβ signaling in stromal cells, facilitated T-cell penetration into the centre of tumors, and provoked vigorous anti-tumor immunity and tumor regression.^42^ It is tempting to speculate that a similar approach might prove fruitful in the treatment of HCC.

Overall, our data contribute to a better understanding of the immune components during HCC progression and are especially important in designing novel immune-based therapeutic strategies to improve treatment for HCC patients. Future efforts will focus on translating the immunomodulatory role of α-TGF-β to achieve a prolonged HCC tumor cell eradication and determining reliable biomarkers to identify patients who will benefit most from such treatment.

## Materials and methods

### Procured HCC samples

Patient samples including HCC (*n* = 98) and matched cirrhotic tissue pairs (*n* = 78), and a cohort of metastatic HCC specimens (*n* = 99) were procured through Folio Biosciences (Powel, OH). Samples were collected with appropriate informed consent from various institutions under protocols approved by Quorum IRB, an appropriately constituted research ethics board as required by regulation, including but not limited to, the ICH Guidelines for Good Clinical Practice, U.S. Food and Drug Administration (21 CFR Parts 50 and 56). All samples were provided as formalin-fixed, paraffin-embedded (FFPE) blocks along with tumor grade, disease stage, and viral status information (Table 1, Supplementary Table S1 and S3). All tissues were assessed utilizing Hematoxylin and Eosin (H&E) staining. RNA was extracted from all tissues and used for Access RNA-Seq.

### In vivo STAM™ mouse model

The STAM™ (NASH-HCC) murine model was generated by SMC Laboratories, Inc., Japan, following a previously established method (http://www.smclab.co.jp/service/nash-hcc.html). At 17 weeks, after the mice had developed HCC, animals were randomly assigned to groups (*n* = 15 mice) and treated with either a vehicle control or TGF-β neutralizing antibody; 1D11 (I.V. injection at 10 mg/kg on the 1st day of treatment, and for remaining doses I.P. injections at 10 mg/kg twice a week), for a total of 6 weeks. Tumors were harvested as individual nodules per liver at the end of the study and were matched with a paired adjacent non-tumor specimen.

### Sirius Red staining and assessment of fibrosis area

Liver snippets were fixed in formalin and embedded in paraffin. Sections were cut from the paraffin block at 4 µm, then deparaffinized and rehydrated in serial dilutions of ethanol. The sections were then immersed in 0.03% PicroSirius Red solution (cat# 1A-280, Waldeck) for 1 h, then dehydrated and cleared with a graded series of ethanol and xylene. The stained sections were mounted with Entellan New (Merck Millipore, Billerica, MA) and observed with a light microscope (Leica Microsystems, Buffalo Grove, IL). After a quick examination of slide quality, 5 fields in the pericentral region (i.e., the area around central veins) were selected and photographed at 200× magnification. The images were imported into ImageJ (National Institute of Health) and processed to de-noise and extract positive areas above a threshold level. The Sirius Red positive area (fibrosis area) was calculated by dividing the positive area (pixels) by the total area (after subtracting the inner space of the cavity) (pixels), and expressed as % positive area.

### CD8/trichrome dual staining

A CD8/Trichrome double-stain was performed on 4 µm FFPE sections. The IHC procedure was performed first using the anti-mouse CD8 hamster monoclonal antibody CD8a:8218 (Genentech, South San Francisco, CA, cat # PRO365247). Processed IHC slides were fixed in Bouin’s fixative at 60 °C for 1 h and were subjected to a routine trichrome stain consisting of Weigert’s hematoxylin, Biebrich scarlet-acid fuchsin, phosphomolybdic-phosphotungstic acid, and aniline blue as described previously.^48^ Slides were dehydrated in increasing ethanol concentration, immersed in xylene, and then coverslipped using a synthetic mounting medium. Whole slide images were acquired with a Nanozoomer XR automated slide scanning platform (Hamamatsu, Hamamatsu City, Shizuoka Pref., Japan) at 200× final magnification. Scanned slides were analyzed in the Matlab software package (version R2017a by Mathworks, Natick, MA) as 24-bit RGB images. Images were processed to determine the percent fibrosis area (trichrome) and the percentage of tumor-infiltrating CD8+ T cells.

Masson’s trichrome detection was used to define fibrotic clusters as dark blue and large contiguous regions by thresholding on blue channel intensity minus the average of red and green channel intensities followed by morphological processing and size filtering. The degree of fibrosis was calculated as a percentage of trichrome positive area (i.e., fibrosis area, sq. µm) relative to tissue area (sq. µm) per nodule.

CD8+ T cells were identified based on intensity thresholding. Differences in spatial localization of CD8+ T cells were determined using the distance from fibrotic deposits per nodule which was used to delineate two major categories of cytotoxic T cells including: trapped (T cells residing within and at the distance of <5 µm from fibrotic clusters), and infiltrated (T cells found >5 µm away from fibrotic clusters). The percentage of trapped and infiltrated T cells was reported per nodule and represents the raw number of T cells in each category (trapped or infiltrated) normalized to its nodule fibrosis area.

### Lysate preparation from FFPE sections

Tissues were scraped from 5 µm thick FFPE sections and digested with Proteinase K mixture at 55 °C for 3–16 h.

### RNA isolation

RNA was extracted from Proteinase K lysates with Roche High Pure FFPE RNA Micro Kit (Roche Diagnostics Corporation, Indianapolis, IN).

### Access RNA-Seq (expression analysis)

Genomic wide transcriptome analysis was run on RNA extracted from FFPE HCC tissue samples. mRNA libraries were prepared using TruSeq RNA Access (Illumina, San Diego, CA). Paired-end 2 × 100 base reads were generated on a HiSeq system (Illumina, San Diego, CA). Reads were aligned to human reference genome using genomic short-read nucleotide alignment program and reads that overlapped gene exonic regions were counted.

### Immunohistochemistry (IHC)

Immunohistochemistry (IHC) was performed on 4 µm thick FFPE tissue sections mounted on glass slides. All IHC steps were carried out on the Ventana Discovery XT automated platform (Ventana Medical Systems, Tucson, AZ). Sections were treated with Cell Conditioner 1, standard time, and then incubated in primary antibody against CD8 clone SP16 (Abcam, cat # ab101500) at 1:100 for 1 h at ambient temperature. Specifically bound primary antibody was detected by the OmniMap anti-rabbit HRP detection kit, followed by ChromoMap DAB (Ventana Medical Systems, Tucson, AZ). The sections were counterstained with Hematoxylin II (Ventana Medical Systems, Tucson, AZ), dehydrated, and coverslipped.

### Cell lines

Human Hepatic Stellate cells (HStec) were obtained from ScienCell Research Laboratories (Carlsbad, CA, cat# 5300). Cells were maintained according to ScienCell in Stellate Cell Media (cat# 5301) supplemented with 2% FBS, stellate cell growth supplement (SteCGS, cat# 5352), and penicillin/streptomycin solution (P/S, cat# 0503) all from ScienCell Research Laboratories. Cells were not used for more than 6 passages after thawing.

### Cell viability assay

Human HStec cells were plated in triplicates at a density of 10,000 cells/well in 96-well plates in Stellate Cell Media as mentioned above and allowed to adhere overnight. The following day, cells were rinsed once with PBS. DMEM media with low glucose supplemented with 0.5% FBS and P/S was added back to the cells with 3-fold serial dilutions of TGF-β neutralizing antibody, clone 1D11.16.8 (GeneTex, cat # GTX14052, CA), starting at 100 µM. Cell viability was assessed 3 days later, using the CellTiter Glo® Luminescence Cell Viability Assay (Promega, cat # G7573, WI). Measurements were read using the Perkin Elmer Envision plate reader. Cell viability measurements were corrected relative to the DMSO control at day 0.

### Protein assays

Human HStec cells were plated at a density of 1 × 10^5^ cells/well in 12-well plates in Stellate Cell Media and allowed to adhere overnight. The following day, cells were rinsed twice with PBS and switched to DMEM media with low glucose with 0.5% FBS and P/S. TGF-β neutralizing antibody was added to cells at 0–20 µg/mL concentrations. After 72 h, supernatant was collected from each well, spun down at 2000 rpm to remove cell debris, and frozen down at −20 °C. Collagen I levels were measured using a type I C-terminal collagen propeptide ELISA kit (Quidel, San Diego, CA, cat # 8003). Supernatants were diluted at 1:30 and ELISA assay were carried out in duplicates. Measurements were read using the Molecular Devices SpectraMax Microplate Reader and protein concentration was measured according to the controls and standards.

### T cell activation and FACS analysis

For T cell activation, total PBMC samples from healthy donors (ALLCELLS, Alameda, CA) were enriched for CD3+ T cell populations using CD3 MicroBeads (Miltenyi Biotec, Auburn, CA, cat # 130-050-101) according to manufacturer’s instructions. Cells were then maintained in OpTmizer CTS T Cell expansion SFM plus T cell expansion supplement (Gibco, Grand Island, NY, cat # A1048501) complete with 5% CTS Immune Cell SR (Gibco, cat # A2596101), 2 mM Glutamine (Gibco, cat # 35050-061) and P/S solution. T cells were activated using Dynabeads Human T-Activator CD3/CD28 beads (Gibco, cat # 11131D). Surface FACS using anti-CD69 (Biolegend, London, UK, cat # 310904) staining was used to detect and confirm the activated state of T cells. TGFβ neutralizing antibody was added to cells within 24 h, and after 72 h incubation, cells were washed and stained with anti-CD4 (BD Biosciences, cat # 560768) and anti-CD8a (eBioscience, Grand Island, NY, cat # 56-0086-82) antibodies and subsequently analyzed with flow cytometry.

### Staining cells for flow cytometry

Activated T cells were washed with PBS then resuspended in Brilliant stain buffer (BD Biosciences, San Jose, CA, cat # 563794) containing FcR blocking buffer (Miltenyi Biotec, Auburn, CA, cat # 120-000-442) and antibodies as recommended by the manufacturer. After 30 min incubation at 4 °C, cells were washed with FACS buffer (PBS plus 0.5% FBS) and resuspended in a 1:1 ratio of FACS buffer and IC Fixation buffer (eBioscience, Grand Island, NY, cat # 00-8222-49). FACS measurements and analysis were carried out using BD LSRFortessa Cell Analyzer and FlowJo_V10 software.

### Statistical analysis of in vitro/in vivo data

Biological experiments and HCC mouse data were analyzed with *t*-tests (2-tailed distribution, unpaired) using GraphPad Prism and Excel software. A *p*-value of <0.05 was considered statistically significant.

### RNA-Seq counts preprocessing

Post data pre-processing and normalization, a total of 176 samples were analyzed (Table 1 and Supplementary Table S1). Genes with less than 10 reads aligned across the entire dataset were filtered out, leaving a total of 15,524 genes for downstream analysis. Pseudo raw gene counts (raw counts + 1) were log transformed and each sample was library size normalized to have a global median of 0. Hereafter this dataset is referred to as HCC1.

### Clustering analysis

The distance between any two samples was defined using their Euclidean distance. The distance between any two genes was defined using their Pearson correlation. Clustering was done (for both samples and genes) using Ward’s agglomerative method.^50^

### Differentially expressed genes (DEGs)

To identify genes that were differentially expressed between the peri- and intra-tumoral samples, a *t*-test was performed for each gene. To identify genes that were associated with stage (T1, T2, T3), analysis of variance (ANOVA) hypothesis testing was performed for each gene using the *genefilter* R package.^51^ All *p*-values obtained from the procedures mentioned above were adjusted for multiplicity using the Benjamini–Hochberg (BH) method^52^ to obtain FDR. For the peri- vs. intra-tumoral comparison, genes with FDR < 12.5% and |log2 (fold-change)| > 1 were called significantly different. For the stage association ANOVA analysis genes with FDR < 25% and log2 (between mean variance) > −3 were selected as significant.

### Gene set enrichment analysis

Gene set enrichment analyses were performed on the Hallmark gene sets [http://software.broadinstitute.org/gsea/msigdb/] using Fisher’s exact test. Obtained *p*-values were adjusted for multiplicity using the BH method. Gene sets with adjusted *p*-value < 10% were called significant.

### Refinement of gene sets

The 89 DGEs with respect to T-cell localization (intra- vs. peri-tumoral) were narrowed down to 23 indication agnostic highly correlated genes as follows: Let ***X***_cor_ denote the Pearson correlation matrix of a given dataset ***X***. Compute a reference correlation matrix, ***R***_cor_ = minimum{***GC***_cor_, ***BUC***_cor_, ***CC***_cor_,, ***KRCCC***_cor_, ***EC***_cor_, ***SC***_cor_, ***RA***_cor_, ***PA***_cor_, ***HCC***_cor_} where the minimum is taken across each cell within the corresponding correlation matrix. Clustering ***R***_cor_ reveals a block of genes (Module 1) that are highly correlated across the indications.

## Electronic supplementary material

Supplementary Information

Supplementary Dataset

## Data Availability

Eight publicly available data sets were used in this manuscript. Six of these TCGA_Bladder Urothelial Cancer (BUC), TCGA_Kidney Renal Clear Cell Carcinoma (KRCCC), TCGA_Esophageal Carcinoma (EC), TCGA_Stomach Adenocarcinoma (AC), TCGA_Rectum Adenocarcinoma (RA), and TCGA_Pancreatic Adenocarcinoma (PA) were obtained from the cancer genome atlas (TCGA) [http://cancergenome.nih.gov/]. The remaining two ACRG_Gastric Cancer (GC) (GSE62254)[40] and Colorectal Cancer (CC) (GSE39582)[49] were obtained from the gene expression omnibus. Additional data from this study are available from the corresponding author upon reasonable request.

## Electronic supplementary material

**Supplementary information** accompanies the paper on the *npj Precision Oncology* website (10.1038/s41698-018-0068-8).

## References

[1] Ferlay J (2015). Cancer incidence and mortality worldwide: sources, methods and major patterns in GLOBOCAN 2012. Int. J. Cancer.

[2] Park JW (2015). Global patterns of hepatocellular carcinoma management from diagnosis to death: the BRIDGE Study. Liver Int..

[3] Mazzanti R (2016). Hepatocellular carcinoma: where are we?. World J. Exp. Med..

[4] Kew MC (2014). Hepatocellular carcinoma: epidemiology and risk factors. J. Hepatocell. Carcinoma.

[5] Kan Z (2013). Whole-genome sequencing identifies recurrent mutations in hepatocellular carcinoma. Genome Res..

[6] Fujimoto A (2016). Whole-genome mutational landscape and characterization of noncoding and structural mutations in liver cancer. Nat. Genet..

[7] Jhunjhunwala S (2014). Diverse modes of genomic alteration in hepatocellular carcinoma. Genome Biol..

[8] Sung WK (2012). Genome-wide survey of recurrent HBV integration in hepatocellular carcinoma. Nat. Genet..

[9] Schulze K (2015). Exome sequencing of hepatocellular carcinomas identifies new mutational signatures and potential therapeutic targets. Nat. Genet..

[10] Li M (2011). Inactivating mutations of the chromatin remodeling gene ARID2 in hepatocellular carcinoma. Nat. Genet..

[11] Totoki Y (2011). High-resolution characterization of a hepatocellular carcinoma genome. Nat. Genet..

[12] Totoki Y (2014). Trans-ancestry mutational landscape of hepatocellular carcinoma genomes. Nat. Genet..

[13] Fujimoto A (2012). Whole-genome sequencing of liver cancers identifies etiological influences on mutation patterns and recurrent mutations in chromatin regulators. Nat. Genet..

[14] Guichard C (2012). Integrated analysis of somatic mutations and focal copy-number changes identifies key genes and pathways in hepatocellular carcinoma. Nat. Genet..

[15] Huang J (2012). Exome sequencing of hepatitis B virus-associated hepatocellular carcinoma. Nat. Genet..

[16] Jiang Z (2012). The effects of hepatitis B virus integration into the genomes of hepatocellular carcinoma patients. Genome Res..

[17] Zimmer V, Lammert F (2011). Genetics and epigenetics in the fibrogenic evolution of chronic liver diseases. Best Pract. Res. Clin. Gastroenterol..

[18] Lee J, Kim Y, Friso S, Choi SW (2016). Epigenetics in non-alcoholic fatty liver disease. Mol. Asp. Med..

[19] Wheeler DA (2017). Comprehensive and integrative genomic characterization of hepatocellular carcinoma. Cell.

[20] Kalluri R, Neilson EG (2003). Epithelial-mesenchymal transition and its implications for fibrosis. J. Clin. Invest..

[21] Lou Y., Diao L., Cuentas E. R. P., Denning W. L., Chen L., Fan Y. H., Byers L. A., Wang J., Papadimitrakopoulou V. A., Behrens C., Rodriguez J. C., Hwu P., Wistuba I. I., Heymach J. V., Gibbons D. L. (2016). Epithelial-Mesenchymal Transition Is Associated with a Distinct Tumor Microenvironment Including Elevation of Inflammatory Signals and Multiple Immune Checkpoints in Lung Adenocarcinoma. Clinical Cancer Research.

[22] Chen L (2014). Metastasis is regulated via microRNA-200/ZEB1 axis control of tumour cell PD-L1 expression and intratumoral immunosuppression. Nat. Commun..

[23] Robinson MW, Harmon C, O’farrelly C (2016). Liver immunology and its role in inflammation and homeostasis. Cell. Mol. Immunol..

[24] Ilkovitch D, Lopez DM (2009). The liver is a site for tumor-induced myeloid-derived suppressor cell accumulation and immunosuppression. Cancer Res..

[25] Pillarisetty VG, Shah AB, Miller G, Bleier JI, DeMatteo RP (2004). Liver dendritic cells are less immunogenic than spleen dendritic cells because of differences in subtype composition. J. Immunol..

[26] Portolani N (2006). Early and late recurrence after liver resection for hepatocellular carcinoma: prognostic and therapeutic implications. Ann. Surg..

[27] Colagrande S, Regini F, Taliani GG, Nardi C, Inghilesi AL (2015). Advanced hepatocellular carcinoma and sorafenib: diagnosis, indications, clinical and radiological follow-up. World J. Hepatol..

[28] El Khoueiry, A. B. et al. Phase I/II safety and antitumor activity of nivolumab in patients with advanced hepatocellular carcinoma (HCC): CA209-040. in *Proc. ASCO Annual Meeting* (ASCO Meeting Library, 2015).

[29] Sangro, B. et al. Safety and antitumor activity of nivolumab (nivo) in patients (pts) with advanced hepatocellular carcinoma (HCC): interim analysis of dose–expansion cohorts from the phase 1/2 CheckMate-040 study. in *Proc. ASCO Annual Meeting* (ASCO Meeting Library, 2016).

[30] Melero, I. et al. Nivolumab dose escalation and expansion in patients with advanced hepatocellular carcinoma (HCC): the CheckMate 040 study. in *Proc. ASCO Annual Meeting* (ASCO Meeting Library, 2017).

[31] Crocenzi, T. S. et al. Nivolumab (nivo) in sorafenib (sor)-naive and -experienced pts with advanced hepatocellular carcinoma (HCC): CheckMate 040 study. *J. Clin. Oncol.* **94**, 10–12 (2017).

[32] Subramanian A (2005). Gene set enrichment analysis: a knowledge-based approach for interpreting genome-wide. Proc. Natl. Acad. Sci. U.S.A..

[33] Liberzon A (2015). The Molecular Signatures Database. Cell Syst..

[34] Luke JJ, Bao R, Spranger S, Sweis RF (2017). Correlation of WNT/β-catenin pathway activation with immune exclusion across most human cancers. J. Clin. Oncol..

[35] Sachdeva M, Chawla YK, Arora SK (2015). Immunology of hepatocellular carcinoma. World J. Hepatol..

[36] Hegde PS, Karanikas V, Evers S (2016). The where, the when, and the how of immune monitoring for cancer immunotherapies in the era of checkpoint inhibition. Clin. Cancer Res..

[37] Tumeh PC (2014). PD-1 blockade induces responses by inhibiting adaptive immune resistance. Nature.

[38] Kalluri R (2016). The biology and function of fibroblasts in cancer. Nat. Rev. Cancer.

[39] Aerts M, Benteyn D, Van Vlierberghe H, Thielemans K, Reynaert H (2016). Current status and perspectives of immune-based therapies for hepatocellular carcinoma. World J. Gastroenterol..

[40] Cristescu R (2015). Molecular analysis of gastric cancer identifies subtypes associated with distinct clinical outcomes. Nature.

[41] Guinney J (2015). The consensus molecular subtypes of colorectal cancer. Nat. Med..

[42] Mariathasan S (2018). TGFβ attenuates tumour response to PD-L1 blockade by contributing to exclusion of T cells. Nature.

[43] Meng X, Nikolic-Paterson DJ, Lan HY (2016). TGF-β: the master regulator of fibrosis. Nat. Rev. Nephrol..

[44] Salama ZA (2016). Losartan may inhibit the progression of liver fibrosis in chronic HCV patients. Hepatobiliary Surg. Nutr..

[45] Klaus A, Birchmeier W (2008). Wnt signalling and its impact on development and cancer. Nat. Rev. Cancer.

[46] Sato R, Semba T, Saya H, Arima Y (2016). Concise review: stem cells and epithelial–mesenchymal transition in cancer: biological implications and therapeutic targets. Stem Cells.

[47] Neuzillet C (2015). Targeting the TGFβ pathway for cancer therapy. Pharmacol. Ther..

[48] Prophet, E. B. Laboratory methods in histotechnology. in *Laboratory Methods in Histotechnology* 132 (American Registry of Pathology, Washington, D.C., 1992).

[49] Marisa Laetitia, de Reyniès Aurélien, Duval Alex, Selves Janick, Gaub Marie Pierre, Vescovo Laure, Etienne-Grimaldi Marie-Christine, Schiappa Renaud, Guenot Dominique, Ayadi Mira, Kirzin Sylvain, Chazal Maurice, Fléjou Jean-François, Benchimol Daniel, Berger Anne, Lagarde Arnaud, Pencreach Erwan, Piard Françoise, Elias Dominique, Parc Yann, Olschwang Sylviane, Milano Gérard, Laurent-Puig Pierre, Boige Valérie (2013). Gene Expression Classification of Colon Cancer into Molecular Subtypes: Characterization, Validation, and Prognostic Value. PLoS Medicine.

[50] Johnson, R. A. & Wichern, D. W. *Applied Multivariate Statistical Analysis* (Englewood Cliffs, NJ, Prentice-Hall, 2014).

[51] Gentelman, R., Carey, V., Huber, W. & Hahne, F. genefilter: Methods for filtering genes from high-throughput experiments. *R Package Version 1.58.1* (2017).

[52] Benjamini Y, Hochberg Y (1995). Controlling the false discovery rate: a practical and powerful approach to multipletesting. J. R. Stat. Soc..

[53] Newman Aaron M, Liu Chih Long, Green Michael R, Gentles Andrew J, Feng Weiguo, Xu Yue, Hoang Chuong D, Diehn Maximilian, Alizadeh Ash A (2015). Robust enumeration of cell subsets from tissue expression profiles. Nature Methods.

