# Supplementary material for: Transcriptomic analysis of hepatocellular carcinoma reveals molecular features of disease progression and tumor immune biology
Source: NPJ Precis Oncol. 2018 Nov 15;2:25. doi: 10.1038/s41698-018-0068-8 (PMC6237857; doi:10.1038/s41698-018-0068-8)
Supplement: Supplementary file 1 — Supplementary Information [file 41698_2018_68_MOESM1_ESM.pdf]

Supplementary Figure S1

A

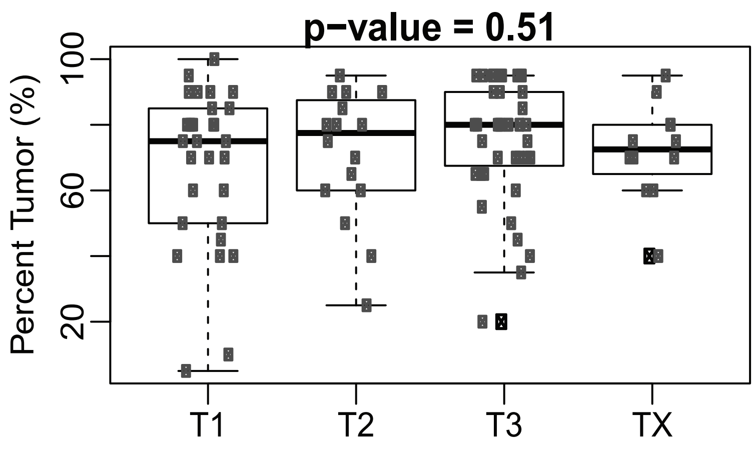

B

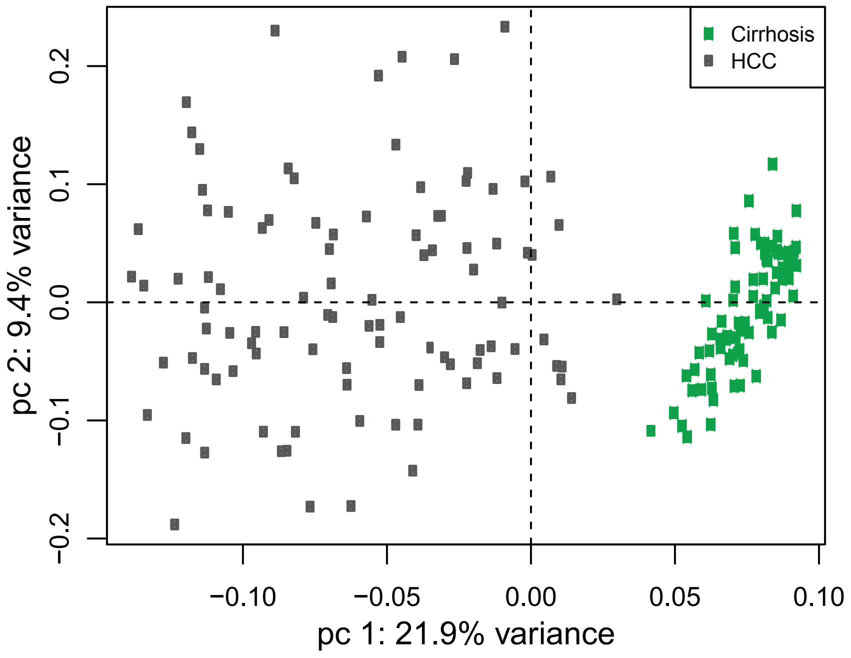

Supplementary Figure S2

A

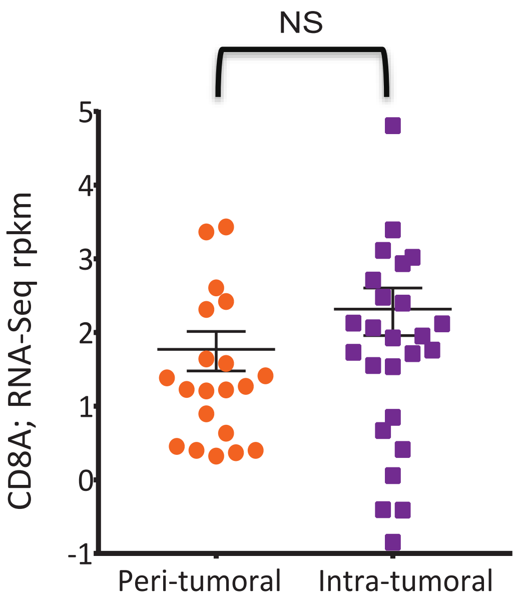

B

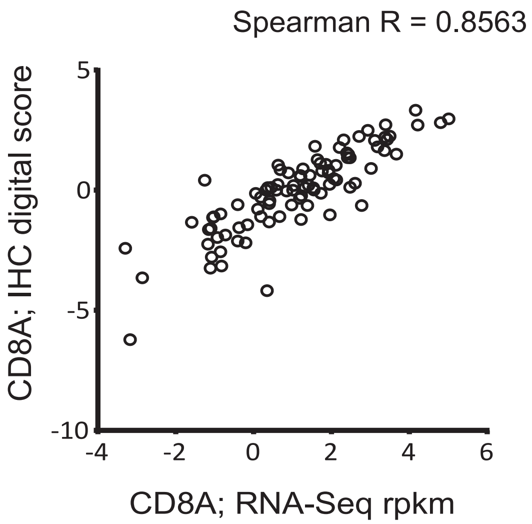

Supplementary Figure S3

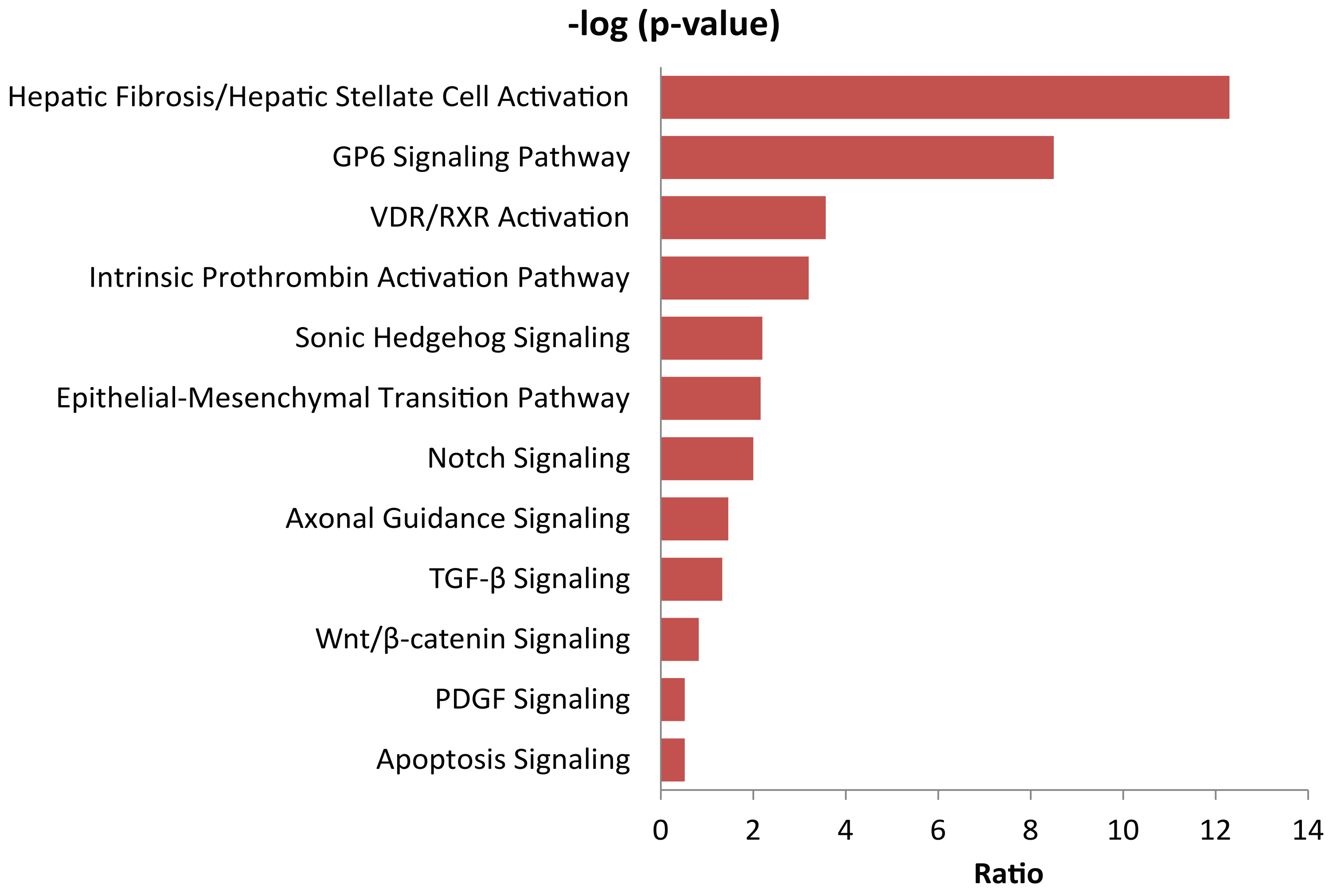

Supplementary Figure S4

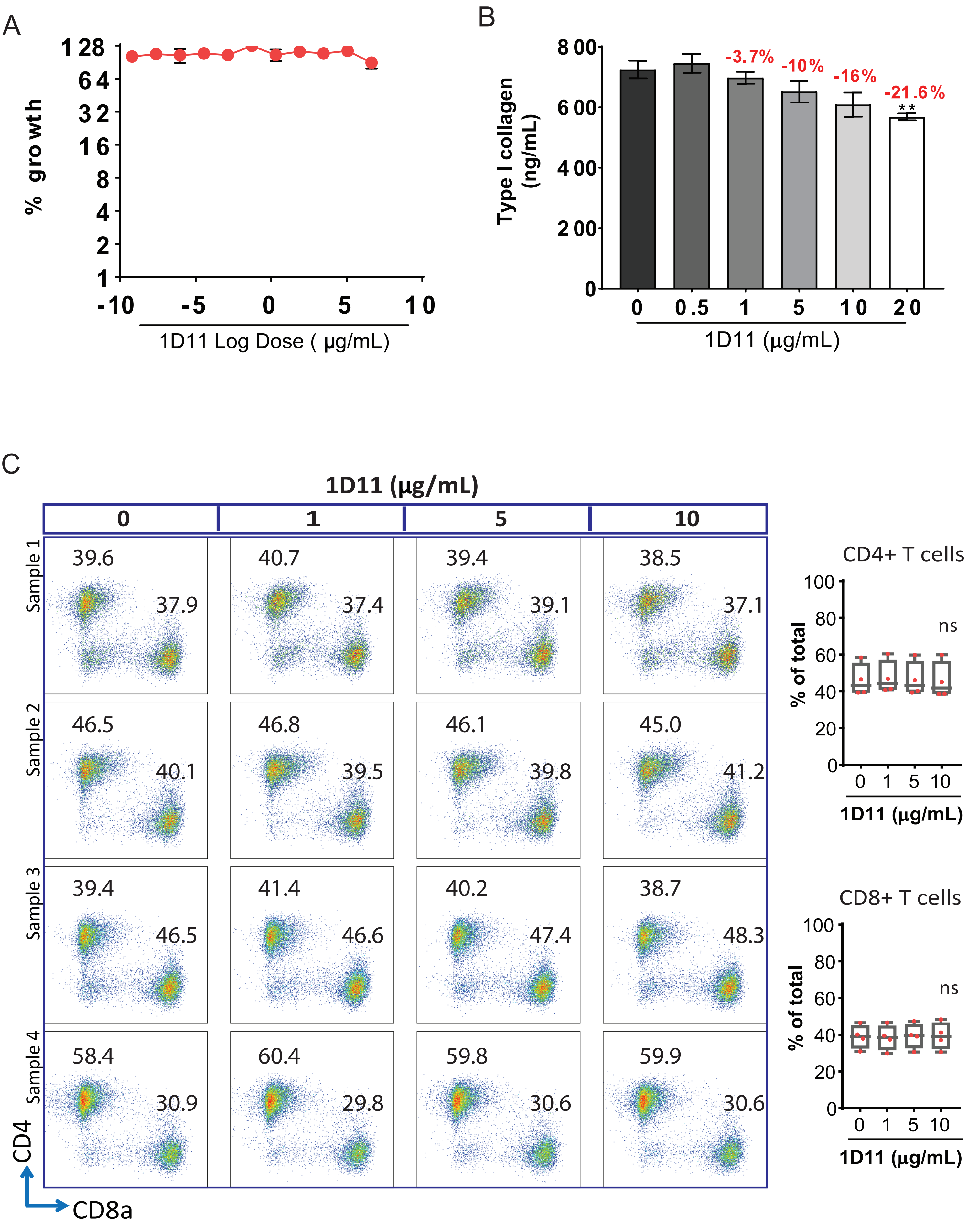

Supplementary Figure S5

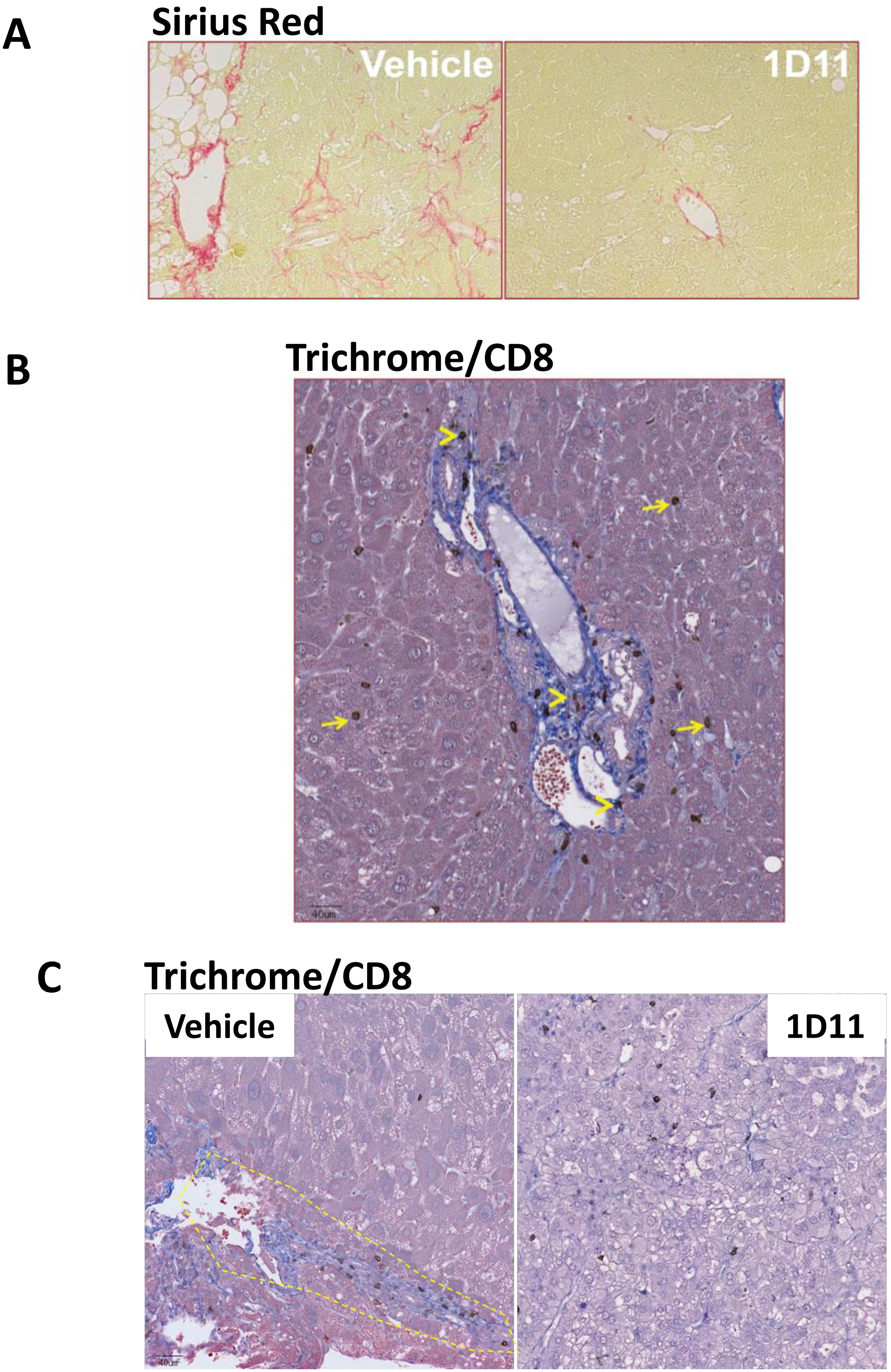

Supplementary Table S1

Cirrhosis	n = 78
**DEMOGRAPHICS**	
**Sex (n (%))**	
Male	64 (82)
Female	14 (18)
**Age (n (%))**	
≤ 50	24 (31)
> 50	54 (69)
**Region (n (%))**	
Asia	78 (100)
**Etiology (n (%))**	
HBV	59 (76)
HCV	2 (3)
Non HBV/HCV	17 (22)

Supplementary Table S2

**Immune phenotype**		
CD8A T cells localization		**n(%)**
Peri-tumoral	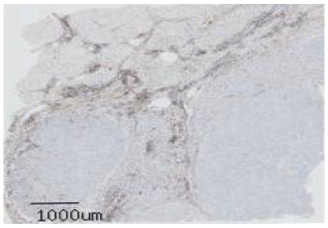	22 (22)
Intra-tumoral & Peri-tumoral	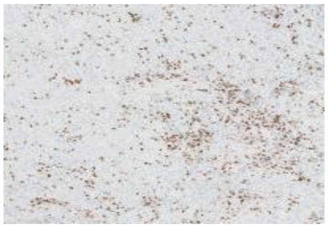	23 (23)
Intra-tumoral	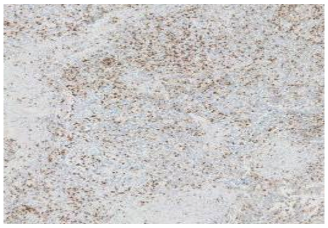	26 (27)
Immunological Ignorant	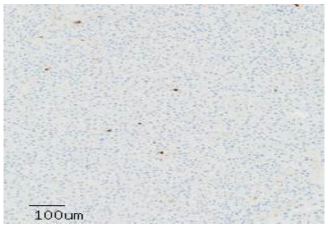	17 (17)

Supplementary Table S3

Patient Samples (Late Stage)	HCC (n = 99)
**DEMOGRAPHICS**	
**Sex (n (%))**	
Male	77 (78)
Female	22 (22)
**Age (n (%))**	
≤ 50	33 (33)
> 50	66 (67)
**Region (n (%))**	
Asia	99 (100)
**Etiology (n (%))**	
HBV	97 (98)
HCV	2 (2)
**TUMOR BURDEN**	
**TNM stage (n (%))**	
**Primary tumor (T)**	
T2	58 (59)
T3	41 (41)
